# Impact of choice of nostril on nasotracheal intubation when using video rigid stylet: a randomized clinical trial

**DOI:** 10.1186/s12871-022-01910-3

**Published:** 2022-11-24

**Authors:** Li-Kuan Wang, Xiang Zhang, Hai-Yin Wu, Tong Cheng, Guo-Li Xiong, Xu-Dong Yang

**Affiliations:** Department of Anesthesiology, Peking University School and Hospital of Stomatology, Beijing, 100000 China

**Keywords:** Video stylet, Nasotracheal intubation, Intubation time, Epistaxis

## Abstract

**Background:**

Patients undergoing oral and maxillofacial surgeries under general anesthesia usually require nasotracheal intubation. When presented with patients with equally patent nostrils, selection of the nostril to use for intubation is an important decision for facilitating intubation. The objective of this trial is to determine whether choice of nostril impacts nasotracheal intubation when using a video rigid stylet in patients undergoing oral and maxillofacial surgery.

**Methods:**

Fifty patients scheduled for elective oral and maxillofacial surgery requiring nasotracheal intubation were randomly allocated into two groups to undergo nasotracheal intubation through the left nostril (Group L, *n* = 25) or the right nostril (Group R, *n* = 25). Intubation was performed by experienced anesthesiologists using a video rigid stylet. The primary endpoint was time to successful intubation, which was defined as the duration from when the tip of the stylet-tube assembly entered the selected nostril to when the tube entered the trachea. Secondary outcomes included: length of time for device insertion; length of time for tube insertion; total success rate; first-attempt success rate; number of intubation attempts; requirement of airway assisted maneuvers; incidence and severity of epistaxis. Intubation-related adverse events were monitored for up to postoperative 24 h.

**Results:**

Median time (interquartile range) to tracheal intubation was 25.3 seconds (20.7 to 27.6) in Group L and 26.8 seconds (22.5 to 30.0) in Group R (median difference (MD) = 1.9; 95% confidence interval (CI) –1.8 to 5.7, *P* = 0.248). Nasotracheal intubation was successful in all patients in both groups and the first-attempt success rates in both groups were similar (Group L: 96% (24/25); Group R: 96% (24/25); relative risk (RR) 1.0; 95% CI 0.9 to 1.1; *P* > 0.999). No significant difference of requirement of assisted maneuvers was noted between the two groups (Group L: 36% (9/25); Group R: 28% (7/25); RR 0.8; 95% CI 0.3–1.8; *P* = 0.544). Furthermore, all patients showed a high quality of visualization of the glottis (Cormack and Lehane Grade I). For safety outcomes, the incidence and severity of epistaxis during intubation was comparable between the two groups. There were no significant differences between the selection of nostrils and intubation-related adverse events up to 24 h after surgery.

**Conclusions:**

When considering which nostril to use for intubation with video rigid stylet, either nostril can be used similarly.

**Trial registration:**

Clinicaltrials.gov. Identifier: NCT05218590.

## Background

Airway management is a highly integral component of general anesthesia. Patients undergoing oral and maxillofacial surgeries under general anesthesia usually require nasotracheal intubation, which allows latitude for operative maneuvering in the face, teeth, mouth, tongue, oropharynx, and neck [1]. Laryngoscopes, including direct and indirect, are the most employed devices for nasotracheal intubation. When presented with patients with equally patent nostrils, selection of the nostril to use for intubation is an important decision for facilitating intubation. Although there is no consensus about which nostril is better for nasotracheal intubation, several studies found that the right nostril may be more suitable for laryngoscopic nasotracheal intubation [2–4]. Two systematic reviews showed that intubation via the right nostril is associated with faster intubation and lower incidence of epistaxis [5, 6].

Laryngoscopic nasotracheal intubation is limited in patients with severely restricted mouth-opening, or microstomia, which is frequently present in oral and maxillofacial patients. Visible rigid stylet is not affected by confined mouth-opening. As such, the visible stylet can be an alternative to the laryngoscope for nasotracheal intubation. Previous studies have shown that optical and video stylets for nasotracheal intubation are safe and effective in patients with either normal anatomy or limited mouth opening [7, 8]. Moreover, it also has been found that the optical stylet was appropriate for awake nasotracheal intubation in patients with predicted difficult airway [9]. For intubation with visible rigid stylet, the tube is first mounted onto the stylet. Then the stylet-tube assembly is inserted from the nostril into the nasal cavity, and into the nasopharynx. When the glottis is viewed, the tube is inserted over the stylet into the trachea. Thus, the maneuverability of the visible rigid stylet is markedly different from laryngoscopic nasotracheal intubation.

The objective of the present study was to determine whether choice of nostril impacts nasotracheal intubation when using a video rigid stylet in adult patients undergoing oral and maxillofacial surgery.

## Methods

### Study design and ethics

This was a randomized, controlled trial. The study protocol was approved by the Ethics Committee of Peking University Hospital of Stomatology, Beijing, China on 01/29/2022 (No. PKUSSIRB-202272018) and registered with ClinicalTrials.gov on 02/01/2022 (NCT05218590). The study was conducted in the Peking University Hospital of Stomatology in accordance with CONSORT guidelines. Written informed consent was obtained from each participant. All methods were performed in accordance with the Helsinki Declaration and relevant clinical trial management regulations of China.

### Participants

During a preoperative visit, we asked patients to occlude the contralateral nostril in the sitting position to self-assess nasal airflow. This method has been verified as useful and accurate for assessing nostril selection for nasotracheal intubation [10]. Patients who were able to breathe clearly and equally through both nostrils were invited to participate in the study. Other inclusion criteria were adult patients aged 18 to 80, American Society of Anesthesiologists (ASA) physical classification I to II, scheduled to undergo elective oral and maxillofacial surgery that required nasotracheal intubation and was expected to last less than 3 hours, and surgery that was anticipated to have extubation performed in the operating room and would not require preventive tracheotomy.

Patients were excluded for the following: (1) history of, or presented with, an anticipated difficult airway. We assessed the airway mainly according to the difficult airway management guidelines of Chinese Society of Anesthesiology [11], which includes predictors of Modified Mallampati classification (III- IV), inter-incisor gap (< 3 cm), thyromental distance (< 6 cm), mandible luxation, head and neck movement, as well as previous history of difficult intubation, and presence of pathologies associated with difficult intubation; (2) required insertion of nasogastric tube; (3) contraindications of nasotracheal intubation; (4) intubation through one nostril due to surgical requirement; (5) presence of severe nasal obstruction, deformities of the nasal cavity, or other serious nasal diseases. Severe nasal obstruction and history of nasal diseases was subjectively reported by the patients themselves. During the preoperative visit, patients were asked if they had difficulty breathing through their nose and/or history of nasal diseases diagnosed by a specialist. All screened patients underwent panoramic radiography, and/or craniomaxillofacial spiral computed tomography (CT), and/or cone-beam CT. Anesthesiologists and maxillofacial surgeons reviewed these radiographs before surgery; (6) history of epistaxis within a month; (7) previous history of nasotracheal intubation, or nasal or laryngeal surgery; (8) language barrier or history of Parkinson disease, dementia, schizophrenia; (9) refusal to sign consent; (10) participated in other clinical studies.

### Randomization and blinding

**Fig. 1:**
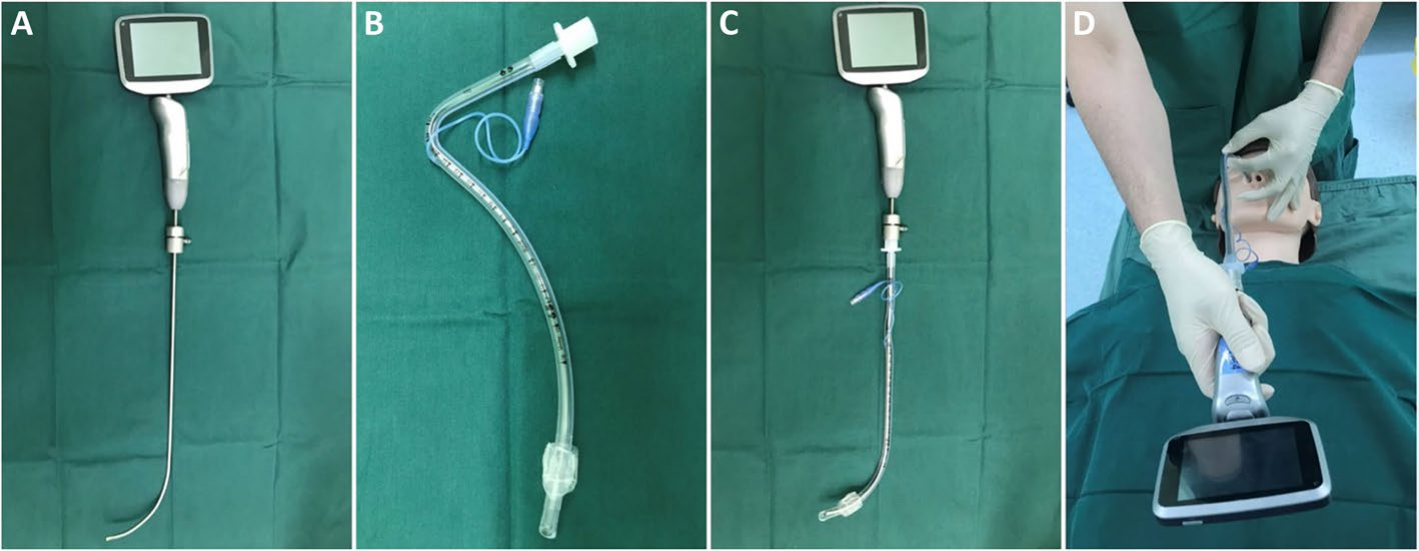
Video rigid stylet and nasotracheal tube. **A** Video rigid stylet; (**B**) preformed double-curved nasotracheal tube; (**C**) before intubation, the nasotracheal tube is preloaded onto the stylet; (**D**) to intubate, the operator’s right hand holds the handle and the left hand threads the stylet-tube assembly into the nostril

Random numbers were produced by the SPSS 21.0 software package (IBM SPSS Inc., Armonk, NY, USA) in a 1:1 ratio by an independent biostatistician and sealed in opaque envelopes. The envelopes were sequentially numbered and opened just prior to anesthesia by an anesthesia nurse who did not participate in the rest of the study. Patients were assigned to nasal intubation through either the left or right nostril (right nostril, Group R; left nostril, Group L). In our study, all intubations were performed using a video rigid stylet (Insight iS3, Shenzhen Insighters Medical Technology, Shenzhen, China), which was patented and approved for human use in 2016 (Fig. 1A). This video stylet has a J-shaped stylet with an adjustable semirigid distal portion. It contains a light source and camera at the distal tip and a color display screen. After intubation, the envelopes were sealed again until the end of the trial. During anesthesia, independent care providers who did not participate in the rest of the study recorded relevant data. The outcome assessors were blinded to the study group assignments. However, it was not possible for the anesthesiologists who performed the intubations to be blinded to the group allocation.

### Anesthesia and intubation

No premedication was given in the general ward. Routine intraoperative monitoring included noninvasive blood pressure, pulse oxygen saturation (SpO_2_), electrocardiogram, oxygen and end-tidal concentration of carbon dioxide, inhalational anesthetic concentration.

**Fig. 2:**
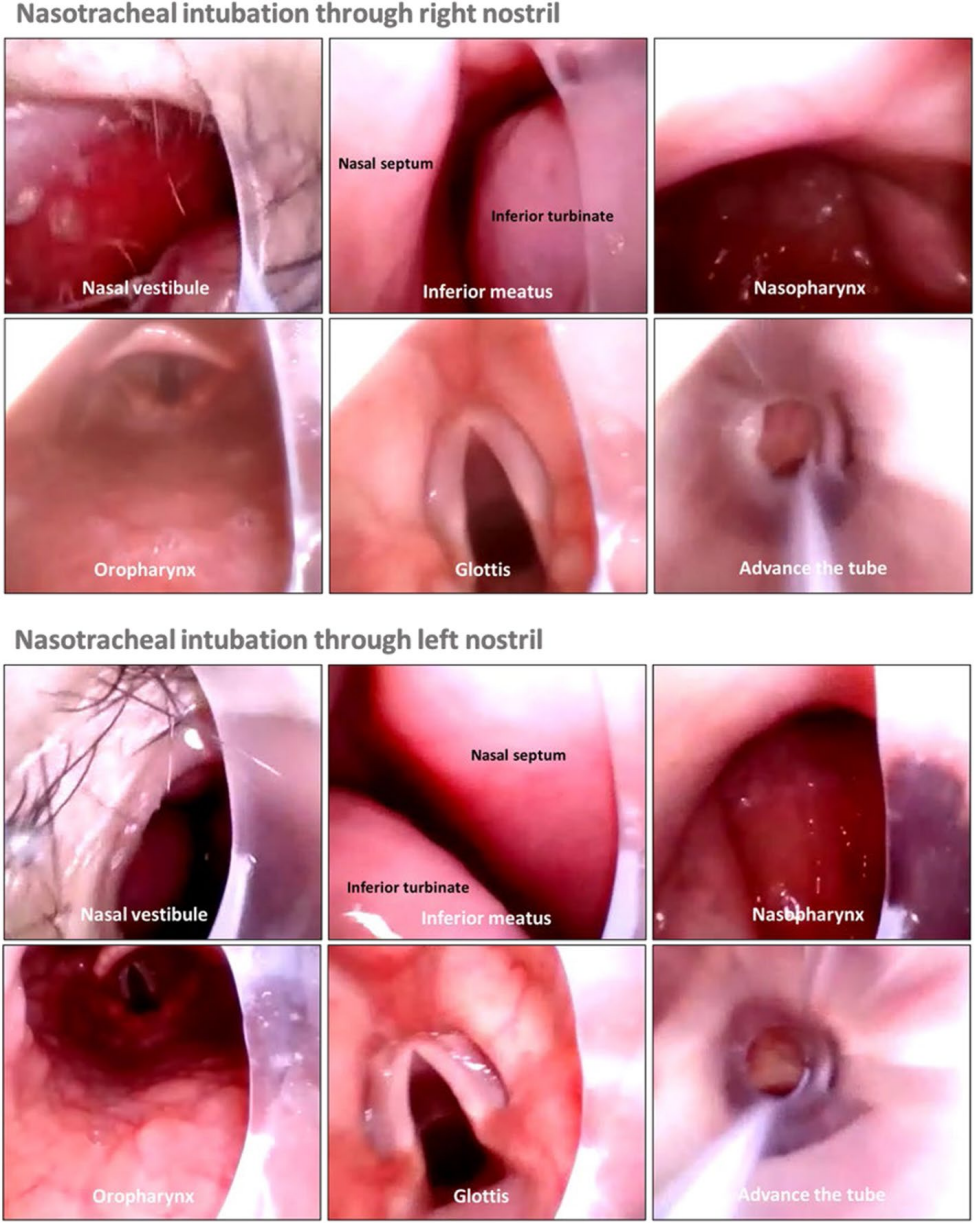
Route of video rigid stylet-guided nasotracheal intubation

Before general anesthesia induction, midazolam and/or dexamethasone were administered intravenously at the discretion of attending anesthesiologist. Anesthesia was induced with sufentanil/remifentanil, propofol/etomidate, and rocuronium/cis-atracurium. Before intubation, topical epinephrine (1:200,000) was applied in the selected nostril. Nasotracheal intubation was achieved using a preformed double-curved nasotracheal tube (Shiley Nasal RAE, Medtronic; Minneapolis, MN, USA) (Fig. 1B), 6.5 mm ID and 7.0 mm ID for female and male patients, respectively. For intubation with the video rigid stylet, an endotracheal tube was lubricated and mounted onto the stylet (Fig. 1C). The operator’s right hand held the handle of the video stylet and the left hand threaded the stylet-tube assembly through the nostril. The patient’s chin was stabilized by the fingers of operator’s left hand (Fig. 1D). The stylet-tube assembly was then inserted into the selected nostril and advanced into the nasal cavity, the nasopharynx, and the oropharynx. After the glottis was exposed and the assembly approached or entered the glottis, the preloaded tube was advanced over the stylet into the trachea (Fig. 2). Successful intubation was confirmed using capnography. General anesthesia was maintained with intravenous infusion of propofol, remifentanil, or inhalational sevoflurane. After surgery, patients were extubated in the operating room.

All intubations were performed by experienced anesthesiologists, who had more than 10 years of experience and had performed hundreds of nasotracheal intubations using video or optical stylets.

### Data collection and outcome assessment

Baseline data included demographic and morphometric characteristics, surgical diagnosis, laboratory workup results, and airway evaluation indicators. Intraoperative data included hemodynamic parameters during intubation, duration of surgery and anesthesia, types and doses of anesthetics, and fluid balance.

Primary outcome was the total time for intubation, defined as the time interval from when the tip of stylet-tube assembly entered the selected nostril to when the tube entered the trachea. Secondary outcomes included: (1) the time for stylet-tube assembly insertion, defined as the time interval from when the tip of the stylet-tube assembly entered the selected nostril to when it accessed the glottis; (2) the time for tube insertion, defined as the time interval from when the stylet-tube assembly accessed the glottis to when the tube was confirmed inserted into the trachea; (3) total success rate; (4) first-attempt success rate; (5) number of intubation-attempts; (6) requirement of airway-assisted maneuvers; (7) incidence and severity of epistaxis. After confirming the location of the tube, an investigator used a fiberoptic bronchoscope to examine the shell of the tube and then examine the posterior pharyngeal wall through the other nostril. No epistaxis was defined as absence of blood on the external surface of the tube and the posterior pharyngeal wall; mild epistaxis was defined as blood observed on the exterior of the tube or posterior pharyngeal wall; moderate epistaxis was defined as pooling of blood on the posterior pharyngeal wall; severe epistaxis was defined as a large amount of blood in the pharynx impeding nasotracheal intubation and necessitating urgent orotracheal intubation [12].

Intubation-related adverse events were monitored for up to postoperative 24 h.

### Statistical analysis

#### Sample size estimation

The primary hypothesis was that the length of time for nasotracheal intubation differs significantly between the two nostrils. A previous study of patients in our center showed the mean (standard deviation (SD)) duration for nasotracheal intubation using an optical rigid stylet was 27 (3) seconds [13]. In that study, the choice of nostril for intubation was based only on the preference of the anesthesiologist (some patients were intubated through the left and others through the right nostril). Theoretically, this intubation duration should be longer than that through the more suitable nostril. Hence, we defined 27 ± 3 seconds as the time required to complete the intubation through the more suitable nostril. The sample size required to detect 10% of changes in intubation time, at a significance level of 0.05 and a power of 90%, is at least 22 patients per group. Thus, we enrolled 25 patients per group. Sample size was calculated with PASS 11.0 software (NCSS Statistical Software, East Kaysville, UT, USA).

#### Data analysis

Data with or without normal distribution were expressed as mean ± SD or median and interquartile range (IQR). Categorical data were presented as a number (%).

For baseline and intraoperative data, quantitative data were compared with *t*-test or Mann-Whitney U test; qualitative data were compared with chi-square test with or without Yates correction, or Fisher’s exact test. Repeatedly measured variables (hemodynamic parameters and SpO_2_) were compared with the general linear model.

Our primary outcome, the total length of time of intubation, was compared with Mann-Whitney U test, with differences between groups expressed as median difference and 95% confidence interval (CI).

For secondary outcomes, quantitative data were compared with *t*-test or Mann-Whitney U test. Qualitative data were compared with chi-square test with or without Yates correction, or Fisher’s exact test. The difference between groups was quantified as the risk ratio (RR), median difference, or mean difference and 95% CI. Safety outcomes were compared with chi-square test with or without Yates correction, or Fisher’s exact test.

A two-tailed *P* < 0.05 was regarded as statistically significant. Statistical analysis was performed with the SPSS 21.0 software package (IBM SPSS Inc., Armonk, NY, USA).

## Results

### Patient recruitment and characteristics

**Fig. 3:**
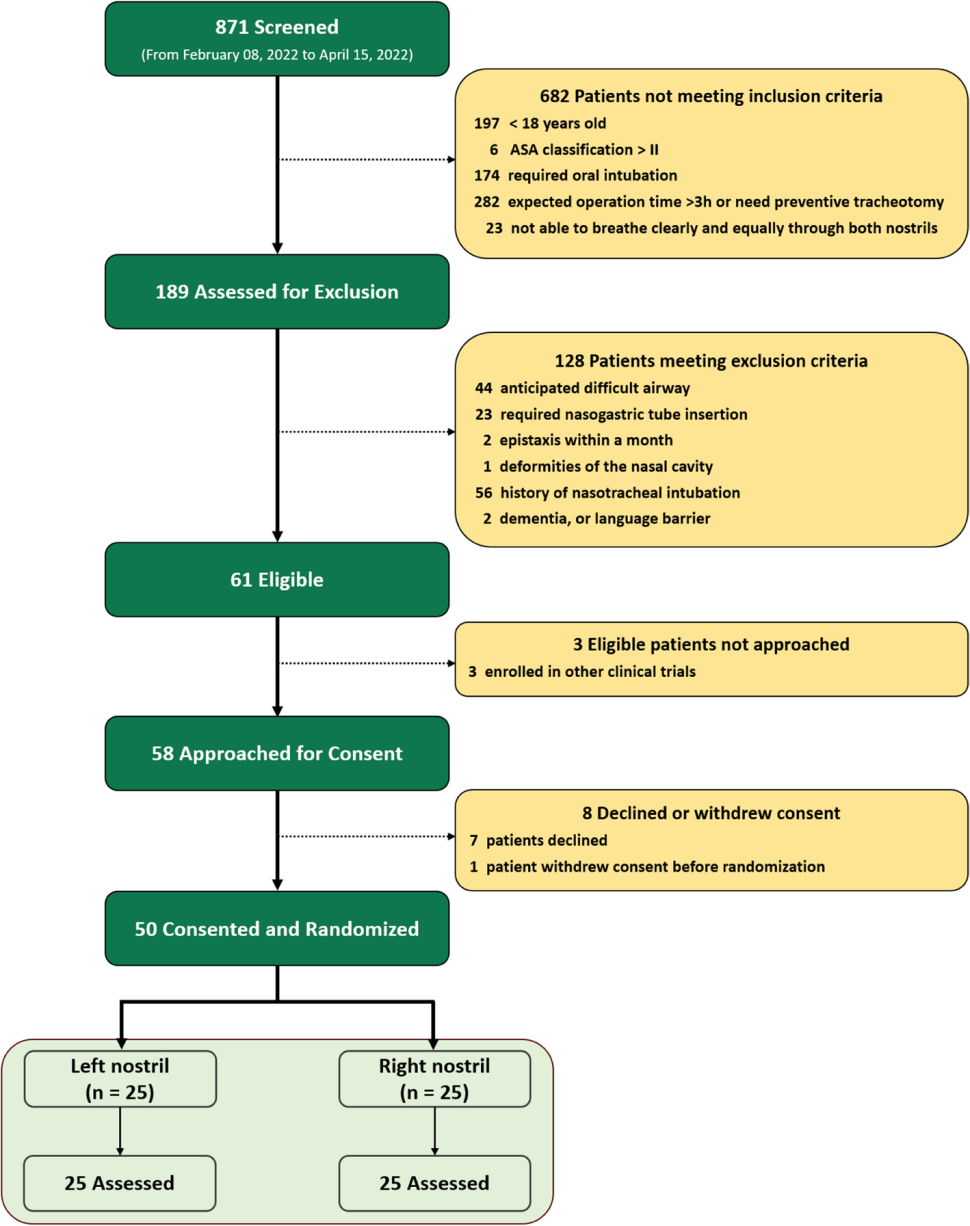
Trial flowchart

**Fig. 4:**
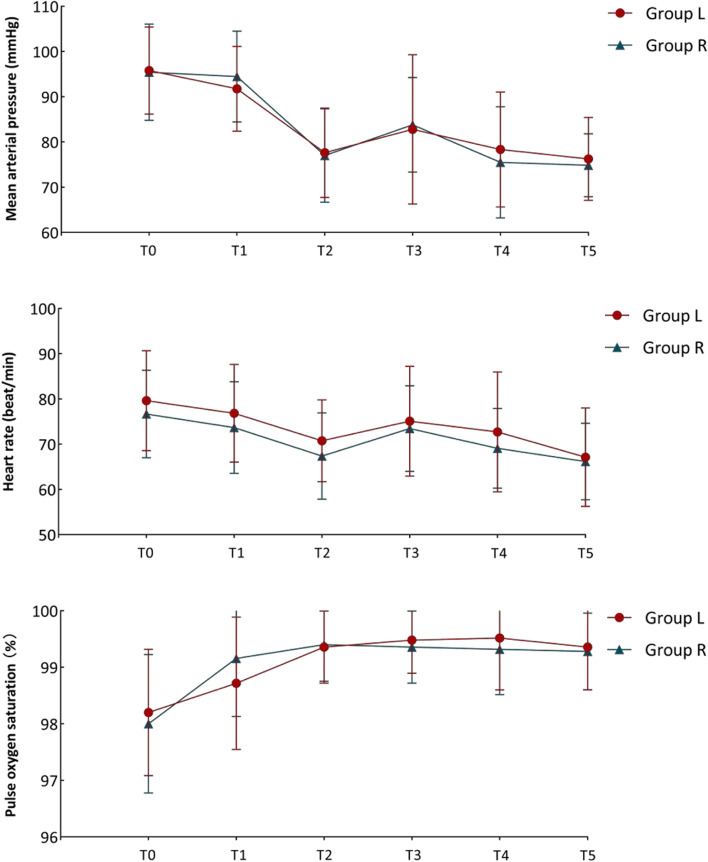
Changes in hemodynamic parameters and pulse oxygen saturation at different time points. T0: Admitted to operating room; T1: immediately before anesthesia induction; T2: immediately before intubation; T3: immediately after intubation; T4: 5 minutes after intubation; T5: immediately before start of surgery

From February 8, 2022 to April 15, 2022, 871 patients were assessed for eligibility (Fig. 3). Of these, 682 patients did not meet the inclusion criteria. A further 189 patients were assessed for eligibility and 128 patients were excluded for various reasons. Of the 61 patients who were eligible, 3 were later excluded for enrollment in other trials and 8 declined participation or withdrew consent. Finally, 50 patients were consented and randomized into two groups, with 25 patients in each group. The two groups were well-balanced regarding the baseline and intraoperative data (Tables 1 and 2). For the hemodynamic parameters and SpO_2_ during intubation, no significant differences were noted between the two groups at any time points (Fig. 4).

**Table 1:** Baseline data

	Group L (***n*** = 25)	Group R (*n* = 25)	***P***-value
Age (y)	40 ± 13	38 ± 13	0.553
Male sex	11 (44%)	9 (36%)	0.564
Height (cm)	169 ± 8	166 ± 8	0.187
Body weight (kg)	64.4 ± 13.4	67.8 ± 14.2	0.392
Body mass index (kg/m^2^)	22.6 (20.2, 27.3)	22.5 (19.8, 26.6)	0.684
ASA classification			0.544
I	16 (64%)	18 (72%)	
II	9 (36%)	7 (28%)	
Laboratory tests			
Hemoglobin (g/L)	141.4 ± 12.0	139.9 ± 12.9	0.676
Platelet (IU/L)	256 ± 56	269 ± 54	0.818
Prothrombin time (s)	11.6 (11.2, 12.2)	11.4 (11.0, 11.8)	0.232
Activated partial thromboplastin time (s)	29.3 (28.2, 31.7)	29.6 (27.6, 30.8)	0.676
Airway evaluation			
Thyromental distance (cm)	8.0 ± 0.8	7.7 ± 0.7	0.203
Inter-incisor gap (cm)	4.5 ± 0.5	4.2 ± 0.6	0.055
Modified Mallampati classification			0.225
I	19 (76%)	15 (60%)	
II	6 (24%)	10 (40%)	

Data are mean ± SD, median (IQR), or n (%)

*Abbreviation:* *ASA* American Society of Anesthesiologists

**Table 2:** Intraoperative data

	Group L (***n*** = 25)	Group R (*n* = 25)	***P***-value
Duration of surgery (min)	36 (29, 43)	33 (26, 37)	0.420
Duration of anesthesia (min)	68 (64, 80)	64 (60, 73)	0.180
Intraoperative medications			
Anesthesia induction			
Midazolam	4 (16%)	7 (28%)	0.306
Etomidate	3 (12%)	2 (8%)	> 0.999
Propofol	23 (92%)	24 (96%)	> 0.999
Dose of propofol (mg)	140.0 (108.5, 170.3)	127.7 (111.6, 154.8)	0.607
Sufentanil	24 (96%)	25 (100%)	> 0.999
Dose of sufentanil (μg)	10 (10, 10)	10 (10, 10)	0.212
Remifentanil	23 (92%)	22 (88%)	> 0.999
Dose of remifentanil (μg)	118.8 (55.6, 146.4)	118.4 (95.6, 158.0)	0.763
Rocuronium	25 (100%)	24 (96%)	> 0.999
Cis-atracurium	0 (0%)	1 (4%)	> 0.999
Medications during anesthesia			
Sevoflurane	2 (8%)	4 (16%)	0.663
Dexamethasone	18 (72%)	20 (80%)	0.508
Total dose of propofol (mg)	535.1 (392.9, 646.2)	440.0 (340.7, 519.7)	0.105
Total dose of sufentanil (μg)	10 (10, 15)	10 (10, 10)	0.143
Total dose of remifentanil (μg)	466.6 (366.5, 598.6)	422.4 (325.9, 488.0)	0.233
Flurbiprofen axetil	24 (96%)	22 (88%)	0.602
Intravenous fluid (ml)	600 (500, 600)	600 (600, 600)	0.342
Estimated blood loss (ml)	20 (15, 30)	20 (15, 30)	0.921
Use of postoperative analgesia pump^a^	10 (40%)	7 (28%)	0.370
Dose of sufentanil (μg)^b^	0 (0, 60)	0 (0, 50)	0.469

Data are median (IQR) or n (%)

*Abbreviation:* *PACU* post-anesthesia care unit

^a^The postoperative analgesia pump, which was prepared with sufentanil and tropisetron 10 mg and diluted with normal saline to 100 ml, was provided for postoperative pain control at a continuous infusion rate of 2 ml/h for 48 h

^b^Dose of sufentanil used in the postoperative analgesia pump

### Efficacy outcomes

Intubation time for Group L and Group R were 25.3 seconds (IQR 20.7 to 27.6) and 26.8 seconds (IQR 22.5 to 30.0), respectively. No significant difference was noted between the two groups (MD = 1.9; 95% CI – 1.8 to 5.7, *P* = 0.248) (Table 3). The times for stylet-tube assembly and tube insertion were also comparable in Group L and Group R (Table 3).

**Table 3:** Efficacy outcomes

	Group L (*n* = 25)	Group R (*n* = 25)	RR or MD (95% CI)	***P***-value
**Primary endpoint**				
Intubation time (s)	25.3 (20.7, 27.6)	26.8 (22.5, 30.0)	1.9 (−1.8, 5.7)	0.248
**Secondary endpoints**				
Stylet-tube assembly insertion time (s)	15.7 ± 5.2	18.7 ± 7.6	3.0 (−0.7, 6.7)	0.113
Tube insertion time (s)	6.5 (5.3, 11.4)	6.6 (4.7, 8.5)	−0.6 (−3.0, 1.3)	0.575
Total success rate	25 (100%)	25 (100%)	–	–
First-attempt success	24 (96%)	24 (96%)	1.0 (0.9, 1.1)	> 0.999
Intubation attempts				> 0.999
1	24 (96%)	24 (96%)		
2	1 (4%)	1 (4%)		
Chin lift	9 (36%)	7 (28%)	0.8 (0.3, 1.8)	0.544
Cormach-Lehane grade				
1	25 (100%)	25 (100%)	–	–

Data are mean ± SD, median (IQR), or n (%)

*Abbreviations:* *RR* relative risk, *MD* median difference or mean difference

Nasotracheal intubation was successful in all patients. The first-attempt success rates in both groups were similar (Group L: 96% (24/25); Group R: 96% (24/25); RR 1.0; 95% CI 0.9 to 1.1; *P* > 0.999). Two patients (1 in the Group L and 1 in the Group R) were intubated on the second attempt due to nasal secretion-stained lens that prevented adequate vision; intubation was successful after wiping the lens. Nine (36%) and 7 (28%) patients in Group L and Group R, respectively required chin lift to assist the intubation. No significant difference was noted between the two groups (RR 0.8; 95% CI 0.3–1.8; *P* = 0.544). Furthermore, all patients showed a high quality of visualization of the glottis (Cormack and Lehane grade I) (Table 3).

### Safety outcomes

Frequencies of epistaxis during intubation were comparable in Group L and Group R (28.0% (7/25) vs. 32% (8/25), *P* > 0.999). Incidences of mild and moderate epistaxis in Group L were 24.0% (6/25) and 4.0% (1/25), and in Group R were 24.0% (6/25) and 8.0% (2/25). No severe epistaxis was observed. There was no significant difference in the severity of epistaxis between the two groups (*P* > 0.999) (Table 4).

**Table 4:** Safety outcomes

	Group L (*n* = 25)	Group R (*n* = 25)	***P***-value
Epistaxis during intubation			> 0.999
None	18 (72%)	17 (68%)	
Mild	6 (24%)	6 (24%)	
Medium	1 (4%)	2 (8%)	
Severe	0 (0%)	0 (0%)	
Adverse events during intubation			
Total incidence	6 (24%)	4 (16%)	0.480
Hypotension^a^	5 (20%)	4 (16%)	> 0.999
Bradycardia^b^	1 (4%)	0 (0%)	> 0.999
Postoperative 24 h adverse events			
Total incidence	9 (36%)	9 (36%)	> 0.999
Continuous epistaxis	3 (12%)	2 (8%)	> 0.999
Pain of the nose	1 (4%)	1 (4%)	> 0.999
Nasal obstruction	2 (8%)	1 (4%)	> 0.999
Sore throat	8 (32%)	6 (24%)	0.529
Severe damage of airway	0 (0%)	0 (0%)	–

Data are n (%)

^a^Defined as systolic blood pressure < 90 mmHg or a decrease of > 30% from baseline

^b^Defined as heart rate < 50 beatmin^−1^ or a decrease of > 30% from baseline

Incidences of intubation-related complications within 24 hours after surgery were not significantly different between the two groups. No severe airway injury or complications occurred (Table 4).

## Discussion

Nasotracheal intubation is an important process in clinical anesthesia for patients undergoing oral and maxillofacial surgery. The visible stylet for tracheal intubation has been employed since the 1980s. Since then, different types of visible stylets have been approved to facilitate tracheal intubation [14]. Slight differences exist between these stylets. Visible stylets can be straight or curved, rigid or semi-rigid. The J-shaped stylet with a semi-rigid adjustable distal portion allows for manipulation depending on airway anatomy, and is thus suitable for nasotracheal intubation. Clinical trials have shown that visible J-shaped stylets are effective and safe tools for nasotracheal intubation in patients with either a normal or difficult airway [7–9, 13]. Results from the present study indicate that the video rigid stylet achieved equivalent length of times for successful nasotracheal intubation through the right or left nostril. Moreover, incidences of epistaxis during intubation and adverse events related to intubation during postoperative 24 h were comparable between patients intubated through either nostril.

When using the laryngoscope, the route of tube insertion via different nostrils is one potential reason for discrepant intubation times. Magill forceps are typically needed for laryngoscopic nasotracheal intubation. Importantly, sufficient length is essential to handle the tube, and the forceps should not occupy too much oral space nor obscure the operator’s field of view [15]. When intubating via the left nostril, the tube is usually displaced to the left along the lateral pharyngeal wall and then placed to the left of the oral cavity. This could make it difficult to view the tube and use the Magill forceps for intubation due to the limited intra-oral field. In addition, the imaging channels of several types of videolaryngoscopes are on the left side of the blade [16]. When placing the videolaryngoscope using the midline approach, insertion of the tube via the right nostril allows leeway for maneuvering of the tube and Magill forceps [17]. Furthermore, when using the videolaryngoscope, nasotracheal intubation can be awkward while holding the tube with Magill forceps on the left side and attempting to view the video screen. However, when using the video rigid stylet, the tube is directly preloaded onto the stylet. Once the glottis is viewed, the tube can be advanced over the stylet directly into the trachea. Therefore, video stylet-assisted nasotracheal intubation does not require an ample intra-oral field and the aid of Magill forceps. The impact of application of Magill forceps was not applicable to video stylet guided nasotracheal intubation.

In addition, with laryngoscopic intubation, the left beveled tip allows the tube to slide easily into the glottis through right side. Conventionally, the default direction of bevel of the tube faces left. The tip of a standard tracheal tube is adapted for the situation that the left-hand laryngoscope used and tracheal tube introduced from the right side [18]. When the tube is introduced from the right nostril, the left-sided bevel can create a relatively sharp tip, allowing easy advancement into the glottis [19]. In contrast, when the tube is introduced from the left side, the tip of the tube with respect to the glottis is rather blunt and the view can be easily obstructed [18]. When using the video rigid stylet for intubation, the tube is loaded on the stylet and navigates with the stylet together to the glottis and then approaches the vocal cord (Fig. 2). Thus, the beveled tip does not affect the entrance of the tube passing the vocal cord. Moreover, when the stylet-tube assembly approaches the glottis, it is easy to adjust the direction of the tube to align with the glottic opening. This also can rid the impact of beveled tip on tube insertion.

The anatomical structures of posterior nasopharyngeal wall can impact nasotracheal intubation. Takasugi et al. [20] retrospectively analyzed CT images and demonstrated that nasopharyngeal anatomical variations were important factors in tube resistance during nasotracheal intubation. When using the video rigid stylet, the tracheal tube is inserted through the nasal cavity with the stylet under direct visualization and the structures of the posterior nasopharyngeal wall can be similarly displayed, regardless of nostril selection (Fig. 2). This reduces the probability of impingement of the tube on the posterior nasopharyngeal wall and shortens intubation time.

Epistaxis is the most common complication of nasotracheal intubation. It can increase the difficulty of airway management and even cause difficult airway, aspiration, and airway obstruction. Epistaxis that occurs with a video stylet can lead to a blood-stained lens, which can obscure the field of view and lead to failed intubation. In two systematic reviews, nasotracheal intubation via the left nostril significantly increased the risk of epistaxis than via the right nostril [5, 6]. It has been thought that the tube bevel’s orientation can impact nasotracheal intubation. Theoretically, with a tube that has a left-sided bevel it is easier to damage the turbinate in the right nostril as well as the septum in the left nostril. The mucosal tissue of the turbinate is thought to be less fragile than that of the septum. This may be one of the reasons for increased incidence of epistaxis when intubating via the left nostril using a laryngoscope. However, although the tube has a bevel, the tip of the tube is not sharp. Therefore, when the tube is adequately lubricated, epistaxis results more likely from impingement of the tube’s tip rather than abrasion of the mucosal tissue of the turbinate or nasal septum. Wang et al. [21] showed that during videolaryngoscope-assisted nasotracheal intubation, incidences of epistaxis were 71% and 12% in patients with and without resistance during intubation, respectively. During laryngoscopic nasotracheal intubation, the tube is blindly advanced through the nasal cavity into the oropharynx. However, the video rigid stylet, allows direct visualization of the anatomy of the intubated route from the nostril to the glottis no matter which nostril is used (Fig. 2). In our study, none of the patients in either group experienced resistance during advancement of the stylet-tube assembly.

Blood pressure has an important effect on severity of nasal bleeding. Boku et al. [2] observed that duration of intubation was significantly longer when using the left nostril. Prolonged intubation can lead to hypercapnia, which can induce elevations in blood pressure and heart rate [22]. In our study, durations of intubation were comparable between the two groups. Moreover, hemodynamic responses during nasotracheal intubation were also comparable between the groups at different time points. These results exclude potential bias from the effects of hemodynamic changes induced by intubation.

In our study, only 3 patients experienced mild epistaxis and no patients had severe epistaxis. These results are lower than those reported in previous studies involving laryngoscopic intubation [2, 23, 24]. Advancement of the stylet-tube assembly under direct vision may decrease the risk of mucosal tearing and thus reduce severity of nasal bleeding. However, as mentioned above, laryngoscopic intubation requires rotation of the tube to align with the glottis. Wang et al. reported that in 26% of patients who underwent laryngoscopic nasotracheal intubation, the tube required adjustment during passage through the nasal cavity, leading to a higher incidence of epistaxis than for patients in whom tubes did not require adjustment [21]. On the other hand, video stylet-guided intubation rarely requires assistance for tube rotation, which may reduce the risk of nasal bleeding.

Epistaxis can be classified as either anterior or posterior, based on location of the ruptured vessels. Kiesselbach plexus on the antero-inferior nasal septum (Little area) is the most common bleeding site [25]. Woodruff plexus, which is on the posterior lateral wall of the inferior meatus of the nasal cavity, has been identified as the common site of posterior epistaxis [26]. It is speculated that intubation-induced nasal bleeding mainly occurs from the Kiesselbach plexus. However, Wang et al. [21] found that the main source of nasal bleeding was due to mucosal damage of the posterior pharyngeal wall. As discussed above, unlike insertion of the tube blindly, intubation using the video stylet permits a clear view of the posterior pharyngeal wall structures regardless of which nostril is used. Moreover, the anteriorly curved stylet surmounts the sharpness of the posterior pharyngeal wall to facilitate advancement of the nasotracheal tube, thus decreasing the risk of intubation-related epistaxis. This has been validated in a previous study that anterior flexion of the stylet tube tip as it approached the curve of the nasopharynx is associated with considerably smoother insertion and reduced bleeding in the nasal cavity [12]. Furthermore, this advantage of the video stylet is not affected by nostril selection.

The primary endpoint of our study was the time for successful intubation. Which primary endpoint to use in studies on intubation techniques remains debatable. In clinical trials on techniques or devices for intubation in patients with difficult or normal airways, intubation time is the most used primary endpoint. The time required for intubation can reflect the efficacy and simplicity of the technique. Certainly, for comparing the efficacy of intubation technique, first-attempt or total success rate may also be logical primary endpoints. However, the use of the video rigid stylet for nasotracheal intubation is a feasible, efficient, and safe technique with a 100% success rate as revealed in published literature [7–9, 13]. Furthermore, in previous studies, nasotracheal intubation with the video rigid stylet has a very high first-attempt success rate of 96.6 to 100% [8, 13]. Even for awake nasotracheal intubation in patients with anticipated difficult airway, a high first-attempt success rate of 92% was shown in previous study [9]. Similarly, in the present study, the first-attempt success rates in the two groups were both 96%. Based on these data, it would be difficult to conduct a trial to investigate the difference on first-attempt or total success rate as a primary endpoint. Another option would be to use incidence of hypoxemia or epistaxis as a primary endpoint. However, in this investigation all intubations were performed by experienced anesthesiologists in patients without any indicators of difficult airways. Thus, the likelihood of hypoxemia was small. As shown in our results, none of the patients experienced hypoxemia during intubation. Furthermore, the incidences of epistaxis in the two groups were 28 and 32%, respectively. Indeed, the incidence of epistaxis is an important endpoint parameter, but to investigate whether choice of nostril impacts epistaxis during video stylet-guided intubation would imply involving a much larger patient population. A randomized trial with a significance level of 0.05 and a power of 0.8 would require a sample size of more than 4114 participants to detect the difference between 28 and 32%. Such a large trial would be difficult to conduct. Moreover, the small difference in incidence of the epistaxis revealed by our data indicates that the impact of nostril choice on the safety of nasotracheal intubation when using video rigid stylet is very small.

The present study has limitations that merit discussion. First, video rigid stylet-guided nasotracheal intubations were performed by experienced anesthesiologists. The procedure requires special training to be competent to use this device. Thus, results from less-experienced anesthesiologists or novice clinicians needs to be evaluated. Second, due to the nature of the intervention, it is impossible to blind the anesthesiologists who performed the nasotracheal intubations; therefore, this could have introduced potential bias. Third, the nasal anatomy of patients was not examined by endoscopy before recruitment. Although nasal endoscopy has many advantages, epistaxis and nasal pain are not rare complaints [27]. This could have affected the outcomes evaluation of our study. Moreover, most previous studies that focused on the impact of choice of nostril on nasotracheal intubation screened participants by assessment of breathing through each nostril [2, 24, 28–30]. Otherwise, this method is the easiest to apply and most employed in clinical settings. Fourth, we only included anesthetized patients without difficult airways.

## Conclusions

The video rigid stylet can be an option in selected cases. Results of our study demonstrated that either nostril is an option when considering which nostril to use for intubation with the video rigid stylet.

## Data Availability

The datasets used and analyzed during the current study are available from the corresponding author on reasonable request.
